# Take Advantage of Glutamine Anaplerosis, the Kernel of the Metabolic Rewiring in Malignant Gliomas

**DOI:** 10.3390/biom10101370

**Published:** 2020-09-26

**Authors:** Filipa Martins, Luís G. Gonçalves, Marta Pojo, Jacinta Serpa

**Affiliations:** 1 CEDOC, Chronic Diseases Research Centre, NOVA Medical School, Faculdade de Ciências Médicas, Universidade NOVA de Lisboa, Campo dos Mártires da Pátria, 130, 1169-056 Lisbon, Portugal; filipa.martins@nms.unl.pt; 2 Instituto Português de Oncologia de Lisboa Francisco Gentil (IPOLFG), Rua Prof Lima Basto, 1099-023 Lisbon, Portugal; martapojo@gmail.com; 3 Instituto de Tecnologia Química e Biológica António Xavier (ITQB NOVA), Avenida da República (EAN), 2780-157 Oeiras, Portugal; lgafeira@itqb.unl.pt

**Keywords:** malignant gliomas, glutamine-glutamate cycle, CNS, cancer metabolism, metabolic adaptation, new metabolic-driven targets

## Abstract

Glutamine is a non-essential amino acid that plays a key role in the metabolism of proliferating cells including neoplastic cells. In the central nervous system (CNS), glutamine metabolism is particularly relevant, because the glutamine-glutamate cycle is a way of controlling the production of glutamate-derived neurotransmitters by tightly regulating the bioavailability of the amino acids in a neuron-astrocyte metabolic symbiosis-dependent manner. Glutamine-related metabolic adjustments have been reported in several CNS malignancies including malignant gliomas that are considered ‘glutamine addicted’. In these tumors, glutamine becomes an essential amino acid preferentially used in energy and biomass production including glutathione (GSH) generation, which is crucial in oxidative stress control. Therefore, in this review, we will highlight the metabolic remodeling that gliomas undergo, focusing on glutamine metabolism. We will address some therapeutic regimens including novel research attempts to target glutamine metabolism and a brief update of diagnosis strategies that take advantage of this altered profile. A better understanding of malignant glioma cell metabolism will help in the identification of new molecular targets and the design of new therapies.

## 1. Malignant Gliomas in Adult, an Overview

Central nervous system (CNS) tumors comprise a complex heterogeneous group of benign and malignant neoplasms from the brain and the spinal cord, having more than 100 histotypes [1]. The classification of CNS tumors was first based on histological variants, being classified according to their morphological features and similarities [2]. Gliomas are brain tumors with glial origin that account for approximately 25.5% of all primary CNS neoplasias, being 80.8% of all the malignant neoplasias affecting the CNS [3]. These neoplasms are characterized by a high mortality rate [3], mainly due to their inaccessible localization in the brain, the high proliferation rate, and infiltrative/invasive capacity [4]. This group embraces several histological entities according to morphological similarities between tumor cells and normal glial cells such as astrocytomas, oligodendrogliomas, and glioblastomas (GBM). Furthermore, these tumors were graded on a malignancy scale, from I to IV: grade I is associated with a better prognosis and lower anaplasia, whilst grade IV is applied to mitotically active neoplasms with the highest degree of anaplasia, being associated with very poor outcomes [2]. However, in 2016, the genetic basis of these tumors was clarified and molecular parameters were also taken into account for World Health Organization (WHO) glioma stratification [1,5]. Hence, the diagnosis and stratification of diffuse gliomas was facilitated by the recognition of isocitrate dehydrogenase 1/2 (*IDH1/2*) mutations and 1p/19q codeletion as principal biomarkers [1]. Mutations in *IDH1/2* occur in the majority of low grade gliomas and secondary GBM, being less frequent in primary GBM [6]. Presently, gliomas are grouped into five main molecular subgroups: GBM *IDH*-wild type, GBM *IDH*-mutant, astrocytoma *IDH*-wild type, astrocytoma *IDH*-mutant, oligodendroglioma *IDH*-mutant, and 1p/19q-codeleted [1,5]. Most importantly, 1p/19q-codeleted and *IDH*-mutant tumors present a better clinical outcome, while GBM *IDH*-wild type presents the worst prognosis, being the most common (50–60%) and the most lethal brain tumors [7,8]. However, it remains highly heterogenic, since it includes patients with a wide range of overall survival (OS), from 1 to 80 months (average OS of 15 months) [9,10,11]. Additional molecular biomarkers are therefore needed to better understand these neoplasms, devise better therapeutic strategies, and increase the accuracy of both glioma diagnosis and prognosis.

## 2. Glutamine and Glutamate Metabolism in the Central Nervous System (CNS)

Glutamine is a non-essential amino acid and the most abundant amino acid in the blood, representing around 20% of the total free amino acid pool [12]. Glutamine plays a role in maintaining pH homeostasis and interorgan nitrogen exchange via ammonia (NH_3_) transport between most proliferating cells, being consequently crucial in the progression of many cancers [13]. As a nitrogen source, glutamine is used as a substrate for nucleotide (purines, pyrimidines, and amino sugars) and nicotinamide adenine dinucleotide phosphate (NADPH) synthesis [14]. As a carbon source, glutamine supplies the tricarboxylic acid (TCA) cycle with oxaloacetate, α-ketoglutarate, and acetyl-CoA, thus being responsible for ATP and macromolecules synthesis, preferentially replacing glucose in certain tumors [15,16,17]. Furthermore, glutamine is a precursor of glutamate, which is necessary for the synthesis of non-essential amino acids and glutathione (GSH), the most important reactive oxygen species (ROS) scavenger and detoxifying agent [18,19].

Glutamate is the most abundant amino acid in the brain, typically present at a concentration of 10–12 μM. However, too much glutamate can be prejudicial, since high levels of glutamate can overstimulate the postsynaptic neurons, leading to CNS damage and causing disturbances such as seizures [20]. Thus, the imbalance of the neuron–glia interactions is extremely important in brain homeostasis.

**Figure 1:**
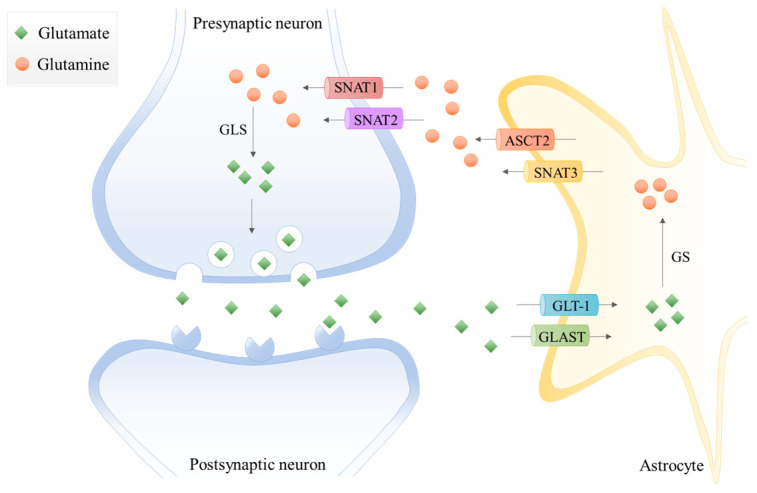
The glutamate-glutamine cycle in a glutamatergic synapse. The released neurotransmitter glutamate is imported by astrocytes through the glutamate transporter 1 (GLT-1) and glutamate aspartate transporter (GLAST). Then, glutamine synthetase (GS) catalyzes the glutamate amidation reaction, generating glutamine using free ammonia. The glutamine is then released from astrocytes via system A amino acid transporter 3 (SNAT3) and alanine/serine/cysteine-preferring transporter (ASCT2) and imported by presynaptic neurons through system A amino acid transporters 1 and 2 (SNAT1 and SNAT2). The glutamine is hydrolyzed to glutamate by glutaminase (GLS), which is packed into synaptic vesicles being sent to the synaptic cleft during neurotransmission. Finally, glutamate is imported again by the astrocytes.

In the last decades, the term “tripartite synapse” was purposed and a new astrocytes function was discovered: the regulation of glutamate levels. In the healthy brain, glutamine is used to synthesize glutamate, which, as mentioned before, is an excitatory neurotransmitter and a precursor of the main inhibitory neurotransmitter γ-aminobutyric acid (GABA). Since neurons lack pyruvate carboxylase (PC) [21], they are incapable of performing de novo synthesis of glutamate or GABA from glucose. Thus, astrocytes and neurons establish a metabolic crosstalk in which astrocytes synthesize glutamine through the glutamate-glutamine cycle that will be later be available to neurons [22]. Basically, astrocytes clear out the glutamate from the synaptic cleft, through the Glutamate Transporter 1 (GLT-1) and Glutamate Aspartate Transporter (GLAST) [23]. Then, the glutamine synthetase (GS) catalyzes the glutamate amidation reaction, generating glutamine. This glutamine is then released from astrocytes via SNAT3 (sodium-coupled neutral amino acid transporter 3), ASCT2 (alanine/serine/cysteine transporter 2), and other transporters, and is imported by presynaptic neurons through SNAT1 and SNAT2 [23]. Afterward, glutamine is hydrolyzed in the neurons by glutaminase (GLS) to glutamate and ammonia. Glutamate is then packed into the synaptic vesicles and sent to the synaptic cleft during neurotransmission. Finally, it is taken up again by the astrocytes [4,24] (Figure 1). Therefore, astrocytes have the pivotal function of removing glutamate from the synapse, mitigating glutamate-induced excitotoxicity.

Extracellular glutamate levels are also regulated by the cystine/glutamate antiporter x_c_^–^system. This glutamate transporter is predominantly expressed in astrocytes, oligodendrocytes, and in some cortical neurons [25]. The x_c_^–^system is pivotal in cell redox homeostasis, once it exchanges glutamate for cystine, which is converted to its reduced form cysteine [18,25]. This is one rate-limiting step of GSH synthesis, since it is a tripeptide composed of glutamate, cysteine, and glycine, with cysteine being the thiol component [19].

In the brain, glutamine synthetase (GS) is expressed in astrocytes, playing a crucial role in nitrogen metabolism [26]. Thus, astrocytes are responsible for ammonia detoxification [27] and the modulation of brain excitability [28] by participating in glutamate and GABA turnover.

Regarding glutaminase (GLS), the two isoforms (GLS-1 and GLS-2) are expressed in neurons and in astrocytes [29]. GLS-1 is the predominant GLS gene expressed in the brain and it encodes two splicing variants, the kidney-type glutaminase (KGA) and glutaminase C isoforms, with its expression modulated by oncogenes such as MYC [30], Rho GTPases [31], and Notch [32]. Through a surrogate promoter usage mechanism [13], GLS-2 encodes two liver-type isoforms, glutaminase 2 (GA) and liver-type glutaminase (LGA), and its expression can be regulated by p53 [33]. Glutaminase isoforms are activated upon low levels of phosphate [23], with GLS-2 being sensitive to lower phosphate concentrations than GLS-1. Furthermore, ammonia activates GLS-2 and inhibits GLS-1 [4].

## 3. Glutamine-Glutamate Relevance in Cancer

Cancer cells undergo metabolic alterations necessary for the acquisition of nutrients, essential for the production of biomass and energy that will sustain the high proliferative rate [34]. Glutamine catabolism is essential for mitochondrial metabolism, since glutamine provides anaplerotic carbons to supply the TCA cycle, accounting for ATP and macromolecules synthesis [35,36,37]. In cancer, glutamine is considered the main TCA cycle supplier upon cancer metabolic remodeling [38]. Increased glutaminolysis rate correlates with carcinogenesis, and its targeting impairs cancer cell proliferation [39,40,41].

The transporters capable of importing glutamine such as ATB^0,+^ (*SLC6A14* gene), SNAT1 (*SLC38A1* gene), ASCT2 (*SLC1A5* gene), LAT1 (*SLC7A5* gene), and LAT2 (*SLC7A8* gene) [42] are crucial in cancer metabolic remodeling often being upregulated in tumors [43,44,45,46,47]. Therefore, glutamine transport targeting is currently being addressed in pre-clinical trials. LAT2 inhibition disturbs glutamine import and disrupts chemoresistance [46]. ASCT2 blockage impairs cancer metabolic remodeling, affecting cancer cell survival [47,48,49,50], thus specific inhibitors are under investigation for future clinical application [51]. However, the redundant activity of glutamine transporters [51,52] can be a mechanism of resistance to a glutamine uptake-targeted therapy.

**Figure 2:**
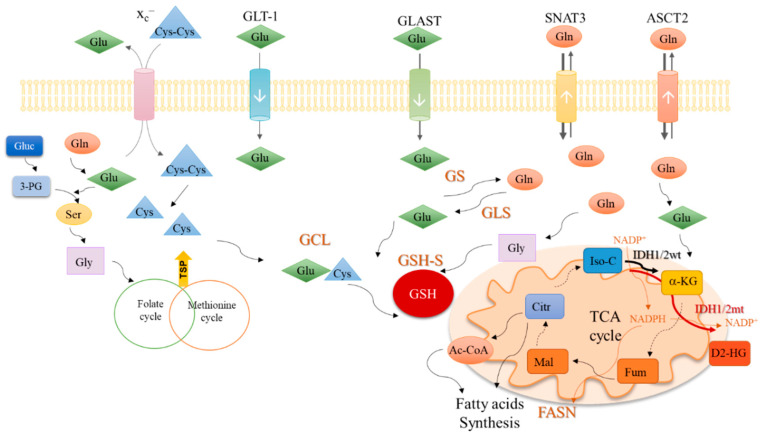
An integrative view of glutamine metabolism. Glutamine (Gln) is a core nutrient in cell metabolism. Gln can be synthesized within the cancer cell, by glutamine synthase (GS) or be taken up from the tumor microenvironment, though different transporters, as SNAT3 and ASCT2. Gln is catalyzed by glutaminases (GLS) enzymes and glutamate (Glu) is generated. Glu can be uptaken from the tumor microenvironment in a process mediated by transporters such as GLT-1 and GLAST. Glu controls the entrance of cystine (Cys-Cys) in the cell, mediated by x_c_^–^ antiporter; Cys-Cys is then converted into cysteine (Cys). Gln-derived Glu is used as a nitrogen source in the synthesis of serine (Ser) from glucose (Gluc)-derived 3-phosphoglycerate (3-PG). Serine (Ser) can be converted into glycine (Gly) that supplies one-carbon metabolism (folate cycle plus methionine cycle) from which cysteine (Cys) is synthesized through the transsulfuration pathway (TSP). Glu, Cys and Gly are the three components of glutathione (GSH), whose synthesis occurs in two steps. In the first step, Glu and Cys are linked by glutamyl-cysteine ligase (GCL) and afterward, Gly is added to the dipeptide Glu-Cys by glutathione synthase (GSH-S). Gln-derived Glu can be converted into α-ketoglutarate (α-KG) and enter the tricarboxylic acids (TCA) cycle. α-KG can also be synthesized by isocitrate dehydrogenase 1/2 wild type (IDH1/2wt) enzymes from isocitrate (Iso-C), with the consumption of NADP^+^ and release of NADPH. NADPH is canalized to other metabolic pathways such as the fatty acid synthesis catalyzed by the fatty acids synthase (FASN). The isocitrate dehydrogenase 1/2 mutant (IDH1/2mt) enzymes further catalyze the conversion of α-KG into the onco-metabolite, 2-hydroxyglutarate (D2-HG), with NADPH consumption. Different Gln-derived TCA cycle intermediates such as fumarate (Fum), malate (Mal), citrate (Citr), and acetyl-CoA (Ac-CoA) can be deviated to supply fatty acid synthesis.

In cytoplasm, glutamine is converted into glutamate (Figure 2) by glutaminase isoenzymes (GLS-1 and GLS-2), which are differently expressed amongst cancer types, presenting an overlapped metabolic function and being pointed as relevant modulators of the clinical outcomes [53]. GLS-2 presents a non-metabolic role related to p53 activation [54] and Snail transcription factor inhibition [55], accounting for GLS-2 classification as a tumor suppressor [53,55]. In malignant gliomas, GLS-2 is commonly downregulated, but GLS-1 is expressed [4,56,57], being pivotal in glutaminolysis, which in turn is crucial for GBM cell survival and tumor growth [58].

Glutamate can be converted into α-ketoglutarate through oxidative deamination by glutamate dehydrogenase 1 (GLDH1) in the mitochondria, or through transamination by amino acid-specific transaminases in the cytoplasm or mitochondria. Aside from α-ketoglutarate, the transamination produces nonessential amino acids such as serine (Figure 2) and aspartate. In cancer, the activation of transaminases is controlled by the MAPK pathway, the main regulator of glutamine metabolism, indicating that glutamine is an important precursor of other amino acids [59] and not only a supplier of the TCA cycle. Glutamine-derived glutamate is the amino group donor in the serine synthesis pathway, in which glucose-derived 3-phosphoglycerate is subject to a step-wise sequence of reactions to give rise to serine [60]. Then, serine can be converted into glycine, under the action of serine hydroxymethyltransferase (SHMT). Next, glycine is canalized to the one-carbon metabolism (folate cycle plus methionine cycle), in which several organic compounds are generated and deviated to supply crucial mechanisms such as the epigenetic modulation (methyl and acetyl groups), nucleotides synthesis, anti-oxidant systems and amino acids, and lipid production (Figure 2) [61,62].

In the mitochondria, α-ketoglutarate enters the TCA cycle and originates other organic compounds such as fumarate, malate, and citrate [63,64] under the action of different enzymes, which are deregulated in cancer. For instance, the malic isoenzymes (ME1 and ME2) are upregulated in different cancer types [65,66,67,68], being observed as pro-tumorigenic [69] and suitable therapeutic targets [65,67]. Interestingly, a direct correlation between ME1 and the pentose phosphate pathway (PPP) was shown, proving that ME1 forms a hetero-oligomer with 6-phosphogluconate dehydrogenase (6PGD), the limiting PPP enzyme, and increases its affinity to the substrate 6-phosphogluconate [70], prompting the deviation of glycolysis intermediates to PPP and promoting the TCA cycle reliance on glutamine. This way, glucose is mainly metabolized in biosynthetic pathways, leaving the bioenergetics role to be played by glutamine. Moreover, citrate synthase (CS) expression is upregulated in cancer upon metabolic stressful conditions such as hypoxia, favoring the metabolic glutamine reliance [71]. Glutamine is also important in biomass production through the above-mentioned intervention in amino acid synthesis and also as a source for lipids, since citrate and glutamine-originated acetyl-CoA is the most relevant lipid precursor [63,72,73], accounting for about 20% of lipogenic acetyl-CoA [72].

The reliance on glutamine metabolism presented by cancer cells can be altered in the GBM-*IDH* mutant, since IDH1/2 mutated enzymes use α-ketoglutarate as a substrate to produce the oncometabolite 2-hydroxyglutarate (D2-HG), therefore a higher commitment of glutamine-derived glutamate is needed to produce α-ketoglutarate that will not be used in the TCA cycle, as will be depicted later on in this review.

### Glutamate-Glutamine Metabolic Remodeling: How Do Gliomas Profit?

In malignant gliomas, the glutamine metabolic remodeling is characterized by the abrogation of GS [74] and the increased expression of glutamine transporters such as ASCT2 [75] and SNAT3 [76]. Therefore, these neoplasms present an increased dependence on the import of glutamine upon the incapacity of producing it [77], acting as ‘glutamine traps’ by importing glutamine from the tumor microenvironment [78].

On the other hand, GLT-1 expression is decreased in glioma cells, impairing the glutamate uptake [79]. Therefore, the increased glutamine uptake and the decreased consumption of glutamate canalizes the excessive glutamate produced by the cell to GSH production and also to the import of cyst(e)ine through x_c_^–^. This phenomenon functions as a mechanism of resistance to radio- and chemotherapy, thereby promoting tumor cell survival [80,81]. Increased GSH levels are correlated with treatment resistance not only in gliomas [82], but also in other types of cancer [19,83]. Moreover, in a study with primary GBM patients performed by Takeuchi et al., strong xCT expression was associated with shorter progression-free survival (PFS) and OS, suggesting a possible role as a predictive factor in GBM [84].

Regarding GLS, low expression of GLS-2 isoforms is a feature of many brain tumors including GBM, anaplastic astrocytomas, and ependymomas [85]. Nevertheless, these tumors express significant amounts of GLS-1 [85]. These facts show that malignant glioma cells are able to fully metabolize glutamine, pointing out this amino acid metabolic route as a core pathway, and consequently a putative target in cancer.

## 4. Glutamine Reliance—A Tool in the Imaging of Gliomas

When planning tumor resection or radiotherapy, it is important to be aware of the precise localization and delineation of the brain tumor, since the maximal possible resection is the most significant treatment-related variable in prognosis improvement [86]. Positron emission tomography (PET) imaging is a clinical tool used for diagnosis, prognosis, and treatment monitoring of diverse pathologies such as cancer, by accessing the tumor uptake capacity of nutrients [87]. This tool is highly sensitive and involves the administration of a specific radiolabeled molecule (radiotracer), usually an analog of glucose 2-[^18^F] fluoro-2-deoxy-d-glucose (^18^F-FDG), which cannot be further metabolized as its uptake is proportional to the reliance of cells on glucose [88], allowing the scanning of the body to detect and measure the emission of positrons [78]. As the brain uptakes high levels of glucose, ^18^F-FDG cannot be used in a clear cut evaluation of brain tumors [89]. However, there is an increasing interest for different tracers to circumvent the limitation of the unclear signal of ^18^F-FDG in the brain. In order to bypass this problem, amino acid traces such as^18^F-fluoro-ethyl-tyrosine (^18^F-FET) or ^11^C-methionine (MET) are already standard of care in neuro-oncology [90,91]. ^18^F-FET allows for a better determination of brain tumor grading, the detection of anaplastic foci, and the evaluation of treatment response [90]. Furthermore, MET shows a good correlation with tumor viability [92], being a powerful tool to distinguish recurrent brain tumor from radiation-resulting necrosis [93].

Given the role of glutamine in the brain, the use of ^11^C or ^18^F radiolabeled glutamine can also be an alternative, as brain tumors show an increased glutamine uptake when compared with the remaining brain tissue [78]. Although 5-[^11^C]l-glutamine has the capacity to be actively transported into glioma cells [94], it has a very short half-life of approximately 20 min, which makes it a tricky clinical tool [95]. In contrast, the glutamine analog [^18^F](2S,4R)4-fluoroglutamine (^18^F-FGln) has a much longer half-life of 110 min, being a good candidate to PET imaging [78,95]. Moreover, animal glioma models and human glioma patients showed high uptake of ^18^F-FGln compared to normal brain tissue in vivo, with a subsequent distinct tumor delineation [96]. This molecule has a higher tumor-to-background signal (of about 4:1 to 6:1) than ^18^F-FDG (approximately 1:1) [96,97]. The uptake of ^18^F-FGln decreased in mice treated with chemo- and radiotherapy, corresponding to a decrease in tumor volume, which indicates its usefulness in monitoring the treatment response in patients [96]. This aspect was reinforced by a recent clinical trial that demonstrated the safety and feasibility of this noninvasive method in different cancer types [98]. Moreover, it was also possible to differentiate high and low-grade gliomas as high-grade gliomas presented elevated ^18^F-FGln, whereas low-grade gliomas were negative for ^18^F-FGln [98].

Another molecule developed for PET imaging was (*S*)-4-(3-[^18^F]fluoropropyl)-l-glutamic acid (^18^F-FSPG). This glutamate analogue is specifically transported via the x_c_^–^ antiporter and shows high and specific accumulation in both animal and human studies of intracranial malignancies [99,100]. This molecule presents low background in healthy tissues and it was indicated as a putative tracer for imaging by a clinical trial with brain tumor patients [100]. Furthermore, some studies have been developed with ^13^N-ammonia, a well-known PET tracer for myocardial blood flow, once it is an important substrate for glutamine synthesis. Since the previously mentioned tracers cannot provide information about de novo synthesis in tumors, ^13^N-ammonia could complement this analysis [101]. For instance, ^13^N-ammonia enables the distinction of brain abscess and necrotic regions in high-grade gliomas [102].

Glutamine metabolism is essential in cancer cells metabolic dynamics, in particular in GBM cells, and its core relevance can be an advantage in cancer management, not only diagnosis, staging, and therapy response monitoring, but also in the outcome prediction and definition of new therapies.

## 5. Malignant Gliomas, a Therapeutic Challenge

### 5.1. Conventional Therapy, Tumor Recurrence, and Therapy Resistance

The treatment of malignant gliomas is dependent on the localization and size of the tumor. Typically, a multidisciplinary approach is taken including surgical removal, followed by adjuvant radiotherapy and chemotherapy. The greater the extent of resection, the longer the PFS and OS will be [103,104]. However, the limit to maximal surgical resection is the potential risk of inducing surgery-related neurological impairment with harmful effects on the patient’s life [105]. Therefore, the complete resection of the tumor is hard to achieve by surgery, once these tumors are invasive and are often in areas of the brain that control speech, motor function, and the senses [104]. Thus, neuro-oncologists hesitate to re-treat local recurrences, since it could mean a loss in neuro-regenerative potential. Regarding radiation therapy (1.8–2 Gy, 30–33 fractions), one limitation is the hypoxic tumor microenvironment, since the presence of oxygen is essential for its effectiveness [106]. In the case of the most aggressive gliomas, GBM *IDH*-wild type and GBM *IDH*-mutant, the gold standard therapy is known as the Stupp protocol and it involves maximal surgical resection followed by radiotherapy with concomitant and adjuvant chemotherapy (temozolomide) [10]. Temozolomide, an oral alkylating chemotherapeutic agent, enters the cerebrospinal fluid with minimal toxicity [106] and acts on cancer cells by inducing DNA damage (double and single-strand breaks). Temozolomide is more effective in tumors exhibiting reduced levels of MGMT (a methylguanine-transferase), due to promoter methylation, which diminishes MGMT capacity to repair temozolomide-induced DNA damages [107,108].

In the last years, tumor-treating fields (TTFields) have gained a lot of attention. This device therapy is based on the delivery of low-intensity alternating electric field to the tumor, interfering with GBM cell division and organelle assembly [109], but the cytotoxic mechanism is not fully understood [110]. In a randomized clinical trial developed by Stupp et al., TTFields treatment in combination with temozolomide resulted in increased OS and PFS [109,111]. Therefore, this method is Food and Drug Administration (FDA)-approved in newly diagnosed and recurrent GBM, however, the neuro-oncology community have been reluctant regarding its widespread adoption [110,112]. Skepticism regarding this device mainly concerns the limited understanding of the mechanism of action, the clinical trial design, and the effect on the patient’s quality of life [112].

Other methodologies have focused on the target of specific pathways that are altered in malignant gliomas. These include the targeting of tyrosine kinase receptors [113], cell cycle control, and molecules related to apoptosis induction [107,114]. Taking advantage of the fact that EGFR amplification is very common in GBM [115,116], several studies have assessed the use of targeting agents against EGFR including monoclonal antibodies, small molecule tyrosine kinase inhibitors, and vaccines [115]. For instance, cetuximab is an anti-EGFR monoclonal antibody already FDA-approved for other cancer types including metastatic colorectal cancer [117]. A phase I clinical trial showed its safety upon osmotic disruption of the BBB with mannitol [118], but its efficacy is still under study in GBM. Even though these strategies may be promising, it has been difficult to apply clinically, due to the heterogeneity of EGFR mutations and even due to compensatory upregulation of other tyrosine kinase receptors [115]. This failure may also indicate the difficulty in finding a targeted therapy to a single pathway altered in this pathology.

Since GBM are extensively neovascularized, an anti-angiogenic strategy has also been considered. Bevacizumab is an anti-VEGF (vascular endothelial growth factor) monoclonal antibody that inhibits the VEGF pathway and it is used in the treatment of some cases of recurrent GBM [107]. Despite several clinical trials showing improvements in PFS, the OS did not show any benefit [119,120], which could be due to a rapid adaptation of the disease to this therapy, or to the fact that the pro-angiogenic mechanisms are not yet fully understood.

As mentioned, the existence of *IDH1/2* mutations is an important marker in brain tumor diagnosis and prognosis. *IDH1* encodes an enzyme that is present in the cytoplasm and peroxisomes, whereas *IDH2* encodes a mitochondrial enzyme. The mutations in these genes are mutually exclusive, being most frequently (>90%) found in *IDH1*. Importantly, this frequency is not altered over cancer progression [121,122]. *IDH1/2* mutated gliomas have a better prognosis due to their slow proliferating rate and aggressiveness [123], again cancer metabolism seems to play a role. Mutated IDH1/2 catalyzes the synthesis of D2-HG from α-ketoglutarate, consuming NADPH [124,125] that will not be available for fatty acid synthesis by fatty-acid synthase (FASN) [126,127], a marker for poor prognosis in *IDH*-wild type gliomas [128,129]. Accordingly, some studies have shown that IDH1 and IDH2 are able to supply fatty acid synthesis by producing citrate from glutamate, which would cooperate with the oncogenic role of FASN [72,124,130,131]. Albeit, this metabolic cooperation is lost in *IDH1/2* mutated tumors. Some studies have been published in order to clarify the role of these mutations in glioma grade progression. The first study showed that the *IDH1* mutation and its inactivation induces hypoxia inducible factor 1 (HIF-1) pathways that are important in tumor growth, inhibition of apoptosis, and cell survival under hypoxic conditions [132]. Simultaneously, another study showed that D2-HG, produced by glioma cells with the *IDH1* mutation, contributes to malignant progression of gliomas [133]. IDH1/2 function can be controlled indirectly by glutamine metabolism, since glutamine-derived glutamate is a precursor of α-ketoglutarate, whose concentrations will determine the rate of IDH1/2 activity. Indeed, cancer cells in certain metabolic conditions such as hypoxia, rather use glutamine-derived compounds in lipogenesis than the preferential IDH1 pathway resulting compounds [72,134,135]. Thereby, patients with IDH-mutated GBM may benefit from glutamine metabolism targeting, since it will efficiently disrupt de novo lipids synthesis, crucial to sustaining cancer cell proliferation and tumor growth [136].

Afterward, another relevant study showed that *IDH1* and *IDH2* mutations reduced α-ketoglutarate and accumulated D2-HG, leading to genome-wide histone and DNA methylation alterations [124]. The metabolite D2-HG binds to the same space occupied by α-ketoglutarate in the active site of histone demethylases, this way inhibiting histone demethylases and the TET family of 5-methylcytosine (5mC) hydroxylases [137]. These alterations, resulting from *IDH1* and *IDH2* mutations, could contribute to gliomagenesis through modifying epigenetic control and potentially the fates of stem or glioma progenitor cells [138]. Then, Lu and colleagues showed that D2-HG-producing *IDH* mutants could prevent the histone demethylation required for cell differentiation for lineage-specific progenitor cells to differentiate into terminally differentiated cells [139]. The *IDH1* mutation remodels the methylome of gliomas, determined as Glioma CpG island methylator phenotype (G-CIMP), which is a powerful factor in tumor pathology [140,141]. G-CIMP was also found in GBM, and was associated with *IDH1* somatic mutations and pro-neural GBM subtype, with these patients having a relatively favorable prognosis [142].

GBM are highly heterogeneous [106], which is one of the hurdles in the management of these neoplasms. Moreover, since the surgical resection is hardly complete, infiltrating tumor cells remain in the brain, leading to later recurrence. Since these treatments are not effective enough, it is necessary to develop new strategies that are able to overcome the main anatomical barrier, the blood brain barrier (BBB). The BBB is a special vessel structure that is composed by three cellular elements of the brain microvasculature: endothelial cells; astrocyte end-feet, and pericytes [143]. BBB tightly regulates the CNS in order to avoid neuroinflammation and neurodegeneration, but allows the entry of innate immune cells [144]. For that reason, in the last years, immunotherapy has gained attention in the field of brain tumors. The fact that activated T cells are capable of entering the brain across the BBB [145] has originated several approaches related to T-cell function including the use of vaccines, adoptive cell transfer (ACT), and immune checkpoint inhibitors [144]. Chimeric antigen receptor (CAR)-T-cell (CART) therapy is the most recent immune-based strategy to treat cancer, and it has presented promising results in malignant glioma models [146,147] and in patients with recurrent GBM [148]. However, most studies show that, despite being well tolerated, immunotherapy failed to prolong GBM patient survival [149], perhaps because it focused on T-cells, which are sparse in GBM [150,151,152]. More recently, an association between the IDH1/2 mutations profile and the macrophagic subsets in GBM has pointed to macrophages and microglia as useful players in new immunotherapeutic approaches [153].

Nevertheless, metabolic fitness is the basis of cancer cell survival, thus the metabolic drift through which cancer cells undergo for sure encloses important details that may be used to design new and deadly therapeutic strategies.

### 5.2. New Therapies: Targeting the Glutamate-Glutamine Cycle

**Figure 3:**
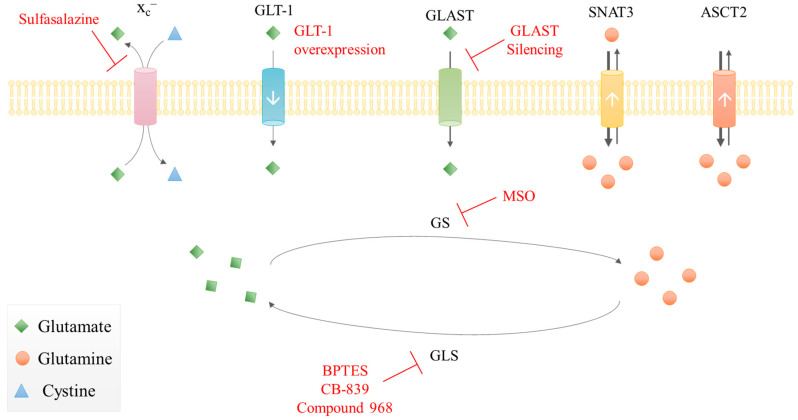
Therapeutic approaches targeting glutamate-glutamine metabolism in malignant gliomas. Summarization of some putative targets and drugs (red) in glioma cells: inhibition of glutaminase (GLS); inhibition of glutamine synthetase (GS); x_c_^–^ inhibition; GLT-1 overexpression; GLAST silencing. White arrows represent the transporters’ expression level in gliomas: down arrow corresponds to lower expression; up arrow is overexpression.

In light of the metabolic remodeling that occurs in malignant gliomas at the level of glutamate-glutamine metabolism, several efforts have been made to identify molecular targets (Figure 3). A study using malignant glioma cell lines by Dranoff et al. showed that the absence of exogenous glutamine did not limit the cells’ proliferation [154,155]. This was due to the existence of two different groups of GBM cell lines, exhibiting low and high GS activity and being respectively “dependent on” and “independent of” glutamine availability in the tumor microenvironment [4]. Rosati et al. also observed this variation of GS levels between tumor samples and found a correlation between low GS expression in tumors and longer OS of GBM patients [156]. Moreover, the GS selective irreversible inhibitor methionine sulfoximine (MSO) demonstrated anti-proliferative activity only on glutamine-independent cell lines, the ones with higher GS activity [154]. However, this compound presents neurotoxicity [157], making its clinical use limited.

The role of GS is also uncertain, whether it acts as a pro- or anti-glioma enzyme. While low GS levels can be beneficial for GBM patients as above-mentioned [156], an experimental study showed that silencing GS potentiated rat C6 glioma cell motility [158], whereas in another study, GS silencing decreased GBM cell line proliferation and colony formation both in the presence and absence of glutamine [159]. Therefore, further studies are needed to elucidate the impact of GS expression on glioma behavior and which compensatory metabolic mechanisms are working on cancer cells. In fact, glutamine is considered as a non-essential amino acid at the human body level, but only certain types of cells are able to produce glutamine (e.g., skeletal muscle cells, lung cells, and adipocytes [160]), being the vast majority of cells dependent on glutamine uptake. Aside from increasing glutamine import [160,161], as GBM cells do [162], some cancer cells overcome the inability to produce glutamine by increasing the import of glutamate, in a p53 dependent manner [163]. A study on GBM showed that GS activity could sustain the glutamine needs of cancer cells, and GBM cells that do not express GS are dependent on the import of glutamine produced by astrocytes [159]. Importantly, the expression of GS in cancer seems to be regulated by oncogenic players such as Myc [164], KRAS, and PI3K [165], indicating that the cancer cells’ genetic profile can limit the expression of GS and consequently their capacity of de novo producing glutamine. A study with several cell lines and tumors showed that GS expression is regulated by promoter methylation and the Myc oncogene prompts the overexpression of GS by promoting the active DNA demethylation [164]. Hence, the explanation for the controversial observations on the contribution of GS for GBM cell survival, can be related to the genetic/signaling/epigenetic profile and the metabolic fitness of the tumor, conditioned by individual and microenvironmental particularities that will differently control the expression of GS.

Apart from the targeting of glutamine and GS, glutamate transport and GLS expression can also be seen as putative targets. Compared to normal astrocytes, glioma explants and cell lines showed decreased glutamate uptake [4] due to decreased GLT-1 expression [79]. Therefore, overexpression of GLT-1 in human GBM cell lines inhibited proliferation and induced apoptosis while suppressing tumor growth in a nude mouse tumor xenograft model [166]. These results suggest that the loss of expression of GLT-1 may be correlated with the aggressive phenotype of GBM. The silencing of GLAST inhibits GBM in vitro cell proliferation and migration, this way potentiating in vivo tumor progression [167]. Moreover, Corbetta et al. observed a significant correlation between moderate/high GLAST expression with lower OS in patients that had received standard post-surgical concomitant radio-chemotherapy, which points GLAST as a putative prognostic marker for GBM [167]. However, the mechanisms by which there is a loss of expression of glutamate transporters in gliomas and its correlation with aggressiveness and molecular subgroups of gliomas is currently unclear.

The x_c_^–^ silencing was also tested, since there is a high expression of this antiporter in GBM cell lines and tumors [168]. Although x_c_^–^ silencing reduced glutamate export, the proliferation of glioma cells was not altered. However, the pharmacological inhibition of x_c_^–^ with sulfasalazine induced a selective apoptotic cell death of GBM cells and in a xenograft model of GBM [169]. This effect can be related to ferroptosis, a new type of cell death that is activated by iron-associated lipid peroxidation, which can be reverted by GSH-dependent glutathione peroxidase 4 (GPX4) [170]. Therefore, the inhibition of x_c_^–^, besides impairing glutamate export, also impairs cystine import and consequently cysteine bioavailability, which abrogates GSH synthesis and allows ferroptosis to occur [171,172,173]. Furthermore, sulfasalazine has been tested in several clinical trials and is indicated as a putative good radiotherapy adjuvant, since their combination increased DNA double-strand breaks and increased glioma cell death [174]. The co-administration of temozolomide and x_c_^–^ inhibitors such as erastin have shown very good anti-cancer results in malignant glioma cell lines in a mechanism involving ferroptosis [175].

Concerning GLS targeting, both the silencing of GLS and its allosteric inhibition with Bis-2-(5-phenylacetamido-1,3,4-thiadiazol-2-yl)ethyl sulfide (BPTES) decreased the proliferation of human GBM cell lines [176]. Other GLS inhibitors were also tested after manipulation of IDH1 R132H, Compound 2 or CB-839 inhibits proliferation preferentially in *IDH* mutated cells, but it has a poor BBB penetration [80], and compound 968 was able to sensitize cancer cells to mTOR-targeted therapies in mouse xenograft models [177].

These different strategies are challenging to apply clinically due to the drugs’ adverse effects, but mainly for the high heterogeneity of these tumors, either genetic or metabolic [9,178,179]. Beyond these limitations, there is the already mentioned major anatomical and molecular obstacle, the BBB, which contributes to brain inaccessibility, making glioma treatment a very challenging issue with often-poor success [180]. A therapeutic approach that we think could surpass these obstacles and still target glutamate-glutamine metabolism is a systemic glutaminase treatment. A similar therapy in acute lymphoblastic leukemia (ALL) to limit the access to asparagine (essential amino acid for certain ALL blasts) using asparaginase, was the first successful metabolism-based therapy, which was introduced some decades ago and is still used in ALL treatment [181]. In malignant glioma therapy, we could take advantage of their reliance on glutamine, decreasing the whole-body glutamine availability with this treatment. Since the other cells retain the ability to synthesize glutamine, this approach would preferentially affect glutamine-dependent gliomas, impairing their metabolism and survival. The stratification of patients based on tumor classification (WHO, 2016) and genetic, histological, and metabolic features would help in the identification of patients that could benefit more from this new therapy.

Tumor metabolism is a complex network of metabolic pathways that share intermediate compounds. The differential consumption of organic compounds allows for the adaptation of tumor cells to the various microenvironments. In the CNS, the glutamine-glutamate cycle occupies a preponderant place in the metabolic adaptation and should undoubtedly be explored in order to take advantage of the metabolic specialization presented by these tumors. As aforementioned, the parallel role of IDH to glutamine metabolism ought to be used in the planning and design of new therapies, since the function of mutated IDH enzymes depends on both α-ketoglutarate produced by IDH wild type isoforms or α-ketoglutarate generated from glutamine-derived glutamate. The election of the most relevant players in glutamine metabolism will help to design new therapies that might deadly disturb GBM cells and impair disease progression. Furthermore, the glutamine dependence of GBM cells appears to be preferentially fulfilled by importing glutamine rather than by synthesizing glutamine [182], thus the transporters mediating the uptake of glutamine across the cell membrane are powerful targets in a strategy aiming to destabilize glutamine metabolism. This way, the levels of glutamine in the tumor microenvironment and in blood circulation are determinant for cancer cell survival, and again, targeting glutamine directly by promoting its degradation before reaching the tumor could be an effective therapeutic approach. Nevertheless, the ability of cancer cells to adapt to new stressful metabolic conditions will be always a hurdle in the success of any metabolism-directed therapy, meaning that the metabolic mapping of cancer cells must be drawn in each particular organ and disease context.

## 6. The Metabolome—A Way of Diagnosis and Prognosis of Gliomas

Since cancer cells undergo metabolic remodeling, metabolic profiling can contribute to better diagnose or follow up the therapy effects and response in gliomas. The cerebrospinal fluid is usually the chosen biological material for metabolome analysis since it may reflect the disease status, despite the collection procedure being invasive and impractical for recurrent testing [183,184].

Nuclear magnetic resonance (NMR) spectroscopy is a highly reproducible method that allows for the detection of radiofrequency signals emitted from the nuclear spins of ^1^H, ^31^P, ^13^C, and ^19^F after exposure to an external magnetic field, enabling the measurement of metabolite concentration [184]. ^1^H-NMR is an important tool to detect both glutamine and glutamate, which have similar spectra and are evaluated together, being a suitable technique to evaluate samples from animal models and human patients with brain tumors [78].

Magnetic resonance spectroscopy (MRS) is an in vivo NMR approach that takes advantage of the specificity of NMR with the spatial localization capabilities of MRI (magnetic resonance imaging) instruments, usually used for diagnosis [185,186]. This approach can enlighten on tumor subtype, grade, and invasion and has been used in the evaluation of gliomas and also in meningiomas and medulloblastomas [187,188]. Compared to ex vivo NMR, MRS has a lower sensitivity and a limited resolution, which makes it difficult to correlate with the ex vivo analysis [184]. A way to overcome this problem is the utilization of hyperpolarized ^13^C compounds. With dynamic nuclear polarization (DNP), it is possible hyperpolarize ^13^C-labeled compounds, increasing 10,000- to 50,000-fold their MR-detectable signal-to-noise ratio [189]. A problem with the hyperpolarized compounds is the short lifetime of the hyperpolarization, which can be of only a few seconds, that hinders a more widespread use [189]. Some preliminary studies were performed to study tumor with *IDH1* mutations resorting to hyperpolarized ^13^C MRS. In a rat bearing GBM tumors with different *IDH1* status, injection of hyperpolarized [1-^13^C] α-ketoglutarate led to hyperpolarized [1-^13^C] glutamate production, with these levels significantly lower in mutant *IDH1* tumors compared with their *IDH1*-wild type counterparts [190].

To obtain a more comprehensive metabolome analysis, NMR is often combined with mass spectrometry (MS) [191,192]. MS allows the elucidation of molecular structures and quantitative analysis of small molecules through the ionization of the sample, leading to a separation according to their mass-to-charge ratio (m/z) [193]. This is a more sensitive technique compared to NMR, requiring a smaller sample size [184], being usually coupled with a chromatograph technique (gas, GC, or liquid, LC). In a study from Dang et al., transfected human glioma cell lines with mutant *IDH1* were metabolically profiled by LC-MS and showed an accumulation of D2-HG, a product of α-ketoglutarate reduction by the IDH1 mutant, both in cellular extracts and in the culture medium, compared to the wild type cells [133]. They also analyzed human glioma samples and observed an increase in D2-HG concentration by 100-fold in tumors containing an R132 *IDH1* mutation compared to *IDH1* wild type tumors. Other studies showed that somatic mutations in *IDH2* (R172 and R140) also increased the D2-HG levels in acute myelogenous leukemia (AML) [194,195]. Hence, IDH1/2 mutations lead to an enzymatic gain-of-function that increases D2-HG levels. Therefore, D2-HG has been considered as an oncometabolite [192] and its production could be an efficient strategy to identify the *IDH1/2* mutated subset of patients with malignant gliomas, as shown by Choi et al., who used MRS to detect D2-HG in 30 glioma patients and proved that this noninvasive diagnostic tool could be powerful to apply clinically [196]. Recurrent *IDH1/2* mutations were detected in gliomas from grade II to IV, showing that IDH1/2 mutated enzymes are needed in gliomagenesis [197,198], hence monitoring D2-HG could be a way of following up the therapy response and early diagnosis of clinical relapse. However, efforts have been made in order to optimize the technical execution of MRS to detect D2-HG in order to obtain clear-cut results [197,198,199].

Metabolomic studies allows for the identification of metabolites that could be evaluated in a clinical context. N-acetyl-aspartate is a derivative of aspartic acid that contributes to energy metabolism and lipid synthesis [200]. *N*-acetyl-aspartate role in the CNS is not clear [201], but it is also advanced as a reservoir of glutamate [201], in this way connected to glutamine/glutamate metabolism. Furthermore, *N*-acetyl-aspartate is considered as a marker of neuronal density and viability, since it decreases with insults to the brain [202]. There is a correlation between the decrease of *N*-acetyl-aspartate concentration and the increasing glioma grade, allowing the differentiation between low and high-grade gliomas [203,204], which makes this a useful prognosis marker [205]. Moreover, an increased *N*-acetyl-aspartate concentration is indicative of a good prognosis [206,207], meaning that cancer cells are not metabolically active and may exhibit lower survival and proliferation capabilities.

Choline containing compounds (e.g., choline, phosphocholine, and glycerophosphocholine) are considered the most important metabolic biomarkers for glioma diagnosis [192]. Choline is an indicator of cell membrane density and integrity, since it is a constituent of the phospholipids of cell membranes [189,193]. In MRS, the resonance of the total free choline in the brain was observed, which also comprises compounds containing choline such as phosphocholine and glycerophosphocholine that are not incorporated into the large macromolecules of the cell membrane [189]. Choline levels are usually elevated in tumors and inflammatory processes [194], revealing an increased cell membrane turnover, since free choline results from the degradation of cell membranes and can be utilized again for membrane synthesis [193]. Higher levels of positive cells for Ki-67 (proliferation marker) have been correlated with increased choline concentrations and with greater glioma malignancy [195], which means that an increased glioma grade shows an increase in choline levels [196,197]. To ameliorate the specificity of MRS, metabolite ratios can be used instead of absolute metabolite concentrations [198]. Indeed, choline/*N*-acetyl-aspartate ratios are truly important as a prognostic indicator in brain tumors [192,199,200].

Another important metabolite is creatine, which results from amino acid degradation in the kidneys and liver and is then transported to the peripheral tissues/organs by blood [189]. Its resonance also contains contributions from phosphocreatine, which functions as a short-term energy reservoir and acts as an effective system buffering cellular ATP levels [201]. Even though creatine may be increased in hypo-metabolic states and decreased in hyper-metabolic states [186], creatine remains in a relatively stable concentration in the brain. Thus, it may be used as a reference for MRS, with levels of other metabolites expressed as a ratio to creatine such as choline/creatine or *N*-acetyl-aspartate/creatine [186].

Myo-inositol is an important osmolyte and substrate for the synthesis of the phosphatidylinositol lipid family, participating in the astrocyte osmoregulatory system where it is synthesized [189]. In the glioma context, MRS detected an increase in myo-inositol levels with the decreased glioma grade, which is useful for the tumor grading evaluation [202].

Another relatively common metabolic change in human gliomas is elevated signals of lactate. The increase in lactate is probably due to anaerobic glycolysis and is not detected in normal brain tissue [202,205]. Lactate may not be directly related to glutamine metabolism, but since glutamine can replace glucose as a TCA cycle supplier, the increased levels of glycolysis-derived lactate can be a way of evaluating the use of glutamine to produce energy by cancer cells. There is a direct correlation between lactate levels and glioma grade [205]. Moreover, lactate is also correlated with necrosis, for instance, due to ischemia. Aside from being a putative diagnosis tool, lactate detection can be used to evaluate a therapy effect. In a study by Li et al., the lactate/creatine ratio decreased with an increase in x-ray irradiation, negatively correlating with the cell death rate [208].

The oncometabolite D2-HG is an independent good prognosis marker. Each grade of malignant glioma has specific MRS features, but overall low-grade gliomas are characterized by a high concentration of *N*-acetyl-aspartate, a low level of choline, and an absence of lactate, whereas a decrease in *N*-acetyl-aspartate and myo-inositol and an elevation of choline are characteristic of high-grade gliomas. Therefore, further research is needed to evaluate and improve the clinical application of these metabolome profiling techniques, which could also be useful in glioma stratification. Nevertheless, it is crucial to do this metabolome profiling in conjugation with molecular features of gliomas, in order to find corresponding and clinically relevant molecular, histological, and metabolic signatures.

There are metabolomic studies indicating that it is possible to find glioma metabolic signatures in peripheral blood. In a study that analyzed 96 serum and 81 corresponding tumor samples by gas-chromatography-time of flight MS (GC-TOFMS) from glioma patients with different grade of GBM and oligodendroglioma, it was possible to distinguish the histological type and tumor grade by the serum and tumor metabolic profile [209]. Tumor tissue from GBM presented higher levels of mannitol and phenylalanine and diminished levels of D2-HG, GABA, creatinine, glycerol-2-phosphate, glycerol-3-phosphate, ribitol, and *myo*-inositol when compared with oligodendrogliomas, while in serum, cysteine was found at higher levels in GBM and lysine and 2-oxoisocaproic acid in oligodendrogliomas [209]. In another study, plasma LC-MS metabolomics discriminated between GBM patients with and without *IDH* mutation, and between high (stage 3 and 4) and low (stage 1 and 2) glioma grade [210]. Presence of the *IDH* mutation led to higher levels of *N*-acetylputrescine, trimethylamine-*N*-oxide, niacin, arginine, glucosamine, and methionine in the plasma [210]. Plasma metabolomics was also used to predict the GBM patient survival, and higher levels of arginine and methionine are correlated with increased probability of survival, while higher levels of kynurenate with decreased probability [211]. An exploratory plasma NMR metabolomics study indicated that the levels of creatine, citrate, glucose, pyruvate, and glutamine can be used to discriminate between primary brain tumor patients (among them GBM patients) and healthy controls [212]. In a different approach in glioma patients, the metabolic profile of arterial blood right upstream of a brain tumor was analyzed and compared with venous blood right downstream of the tumor. It was observed that glioma consume large amounts of *N*-acetylornithine, d-glucose, putrescine, and l-acetylcarnitine and produce l-glutamine, agmatine, and uridine 5-monophosphate [213]. Moreover, the in vivo D2-HG production in patients with the *IDH* mutation was also observed [213].

The relevance of GBM cell reliance on glutamine was reinforced by a new study on GBM orthotopic models, which showed the impact of glutamine dependence in the GBM cell metabolome, presenting a panel of glutamine-derived organic compounds [182] that can be used as a reference to help define GBM metabolic signatures to be correlated to different genetic profiles and clinical outcomes.

## 7. Concluding Remarks

This review highlights the impact of glutamate-glutamine metabolism in the context of malignant gliomas. Since conventional therapy based on surgical removal, radio- and chemotherapy is not effective, new strategies are needed to treat malignant gliomas. Until now, the metabolic-driven therapeutic approaches have been challenging to apply clinically, not being effective thus far. Therefore, there is still an emerging need to test novel metabolism driven strategies that target various aspects of glutamate-glutamine metabolism in malignant gliomas, for instance, the use of a systemic glutaminase treatment.

A metabolism-based multidisciplinary approach including different ‘omics’ should be used to improve glioma diagnosis, prognosis, and follow up. Regarding metabolome-based diagnosis, up to now, there has been a small panel of metabolites identified as helpful for the discrimination of glioma subgroups and therapy response prediction. The accurate stratification of patients is crucial in choosing the best therapeutical protocol, thus reducing the therapy related toxicities and the tumor recurrence. Therefore, the combination of MS and NMR can be applied to explore more signature metabolites of brain tumors.

Taking advantage of the natural features of cancer cells that affect determinant metabolic pathways and profiles such as *IDH* mutations and glutamine-glutamate metabolism can be a valuable opportunity to find effective new strategies that trigger a bottom-up cancer cell death and tumor impairment by targeting the most basal life-sustaining mechanisms and reflecting their disturbance in tumor regression.
